# Topical Anesthesia Offers Sufficient Pain Control for MicroPulse Transscleral Laser Therapy for Glaucoma

**DOI:** 10.1155/2022/6845434

**Published:** 2022-09-21

**Authors:** Belgin Vasella, Marc Töteberg-Harms

**Affiliations:** ^1^University Hospital Zurich, Department of Ophthalmology, Zurich, ZH, Switzerland; ^2^University of Zurich, Medical Faculty, Zurich, ZH, Switzerland; ^3^Augusta University, Medical of Georgia, Department of Ophthalmology, Augusta, Georgia, USA

## Abstract

**Introduction:**

The aim of this study was to evaluate patient pain during and after MicroPulse Transscleral Laser Therapy (TLT) and vision-related quality of life using two different anesthesia protocols: “Topical Plus” anesthesia without standby anesthesia (study group), and analgosedation with standby anesthesia (control group).

**Methods:**

A retrospective, comparative chart review was conducted to evaluate patient pain between the two groups based on an analog pain scale at baseline and postoperatively (1 hour, 6 hours, 1 day, 1 week, and 1 month). Furthermore, vision-related quality of life at baseline was compared at 1 month postoperatively.

**Results:**

Four eyes underwent MicroPulse TLT under Topical Plus anesthesia and 4 eyes underwent analgosedation. The mean age at the time of the MicroPulse TLT was 78.3 ± 6.4 years. In the study group, the reported pain level increased significantly immediately after the treatment (from 0.5 to 2.8, *p*=0.003; mild pain); however, no difference was found at any later time point compared to baseline. The vision-related quality of life was similar in both groups and was not negatively impacted by the MicroPulse TLT at 1 month after the treatment. *Discussion/Conclusion.* The Topical Plus anesthesia protocol provides adequate pain control for the patient to remain comfortable during and post-MicroPulse TLT treatment, with no changes in vision-related quality of life. Although the sample size was small and, thus, the results cannot be generalized, this case series showed that it is possible to perform MicroPulse TLT under topical anesthesia.

## 1. Introduction

Glaucoma is one of the leading causes of irreversible blindness worldwide. Lowering intraocular pressure (IOP) is the only evidence-based treatment for glaucoma [1–4]. Topical glaucoma treatment options are burdensome and often poorly tolerated by patients and associated with compliance and adherence problems [5]. The surgical gold standard is still penetrating, filtration surgery, that is, trabeculectomy or tube shunts [6, 7]. However, several minimally invasive glaucoma surgery (MIGS) procedures have evolved in the last one to two decades [8–10].

Besides penetrating, filtration surgery, and MIGS procedures, MicroPulse Transscleral Laser Therapy (MicroPulse TLT) is another option to treat glaucoma. It is a nonincisional laser procedure facilitated with an 810 nm infrared diode laser (Cyclo G6® Laser System; Iridex, Mountain View, CA, USA). The laser energy is applied transsclerally using the MicroPulse P3® Probe (Iridex, Mountain View, CA, USA). The MicroPulse P3 is a handheld fiberoptic probe that the surgeon sweeps back and forth along the limbus in the superior and inferior hemisphere of the globe which enables laser energy delivery at a predetermined distance from the limbus. The area around the three- and nine-o'clock positions is spared to prevent damage to the long ciliary nerves. MicroPulse TLT can be used in various types and stages of glaucoma [11]. The current evidence shows that MicroPulse TLT provides a superior safety profile than traditional continuous-wave transscleral cyclophotocoagulation (CW-TSCPC) while maintaining a significant IOP lowering effect [12–14].

Patients can expect effective anesthesia for sufficient pain control during the MicroPulse TLT procedure. In addition, sufficient pain control enables precise and correct execution of the sweeping motion by the surgeon. The following options for anesthesia for MicroPulse TLT have been discussed traditionally with patients: (1) subconjunctival anesthesia, (2) subtenon's anesthesia, or (3) a retrobulbar block. Conjunctival bleedings should be avoided due to possible laser energy absorption at the used wavelength, that is, 810 nm; otherwise, limited efficacy by minimizing laser transmission and absorption of the target tissue can result. Hence, the prior administration of a topical vasoconstrictor (e.g., brimonidine 0.2% or phenylephrine 5%) should be considered. In addition, (4) analgosedation (e.g., propofol plus fentanyl or thiopental plus fentanyl) can be offered for MicroPulse TLT. Analgosedation allows for excellent pain control and puts the patient to sleep for a few minutes, sufficient time for the MicroPulse TLT procedure. Furthermore, (5) general anesthesia is an option as well. Analgosedation eliminates the need for a retrobulbar block. For blocks, analgosedation, and general anesthesia, monitored anesthesia care is mandatory, while topical anesthesia including “Topical Plus” can be considered to be performed without monitored anesthesia care.

We recently introduced the “Topical Plus” anesthesia protocol for MicroPulse TLT procedures. It combines topical anesthesia drops and Lidocaine 2% (Xylocaine Gel 2%, Aspen Pharma Schweiz GmbH, Baar, Switzerland) nonalcoholic gel. “Topical Plus” anesthesia or analgosedation for MicroPulse TLT are routinely offered at the University Hospital Zurich.

In our clinical experience using the “Topical Plus” regimen, we have observed that patients complain of minimal discomfort and pain during and after the MicroPulse TLT treatment and usually require minimal to no oral pain medication in the first 24 hours postoperatively. The pain level during the treatment did not increase to the point that the MicroPulse TLT treatment had to be discontinued due to pain. In fact, many patients were relieved having the option to choose “Topical Plus” anesthesia as an alternative to analgosedation, as medical conditions in some patients pose an increased risk for unexpected incidents during analgosedation.

With the “Topical Plus” anesthesia protocol, MicroPulse TLT can be performed with or without standby anesthesia. It can be performed in the operating room, a minor procedure room, or in an ophthalmological examination room.

Topical anesthesia with lidocaine gel is an established form of anesthesia for cataract surgery with proven pain control [15, 16]. Most glaucoma specialists who perform MicroPulse TLT have previously performed CW-TSCPC. CW-TSCPC induces high pain during and after treatment. Thus, most surgeons continued with their anesthesia and pain control protocol when switching from CW-TSCPC to MicroPulse TLT. However, some surgeons who considered MicroPulse TLT to be less painful than CW-TSCPC changed their anesthesia and pain control protocol to account for less pain during MicroPulse TLT.

The aim of this study was to evaluate pain during and after MicroPulse TLT and visual quality of life under the “Topical Plus” anesthesia protocol without standby anesthesia compared to analgosedation with standby anesthesia. We hypothesize that the “Topical Plus” protocol is sufficient to control pain during and after MicroPulse TLT.

## 2. Materials and Methods

This is a retrospective, comparative chart review of patients with open-angle glaucoma treated with MicroPulse TLT at the University Hospital Zurich (USZ), Zurich, Switzerland, between September and October 2021. Data from previous months have not been included, as surgeons changed their treatment protocol for performing MicroPulse TLT; to achieve better success rates, sweep velocity was reduced from 10 seconds per hemisphere to 20 seconds per hemisphere [17–19]. Prior to the study, approval of the protocol by the cantonal ethics commission of Zurich (KEK ZH, BASEC No. 2020–00762) was granted. Furthermore, the study follows the principles of the Declaration of Helsinki. All study subjects gave written informed consent to the study. Both forms of anesthesia were carefully explained and offered to all patients. The choice between the two anesthesia protocols was left to the patient's discretion.

### 2.1. Inclusion and Exclusion Criteria

Patients included in the study had open-angle glaucoma and underwent MicroPulse TLT between 09/01/2021 and 10/31/2021. Patients were excluded from the study if they did not provide their written study-specific consent.

### 2.2. Procedure, Anesthesia, and Postoperative Care

All procedures were performed by one glaucoma specialist (MTH). Patients had either “Topical Plus” anesthesia (study group) or analgosedation (control group) as described below. The “Topical Plus” protocol was as follows:

1. Topical unpreserved tetracaine 1% (Tetracaine 1% SDU Faure, Théa PHARMA S.A., Schaffhausen, Switzerland) eye drops were applied 1 to 3 times to provide more comfort during insertion of an eye speculum.
2. An eye speculum was inserted.
3. Lidocaine 2% gel was applied multiple times over 5 minutes on the eye surface to ensure full coverage of the surface by the gel during the entire 5 minutes. The speculum could be lifted slightly up to give better access for the Xylocaine gel to the fornices including the subtarsal conjunctiva.
4. The speculum was removed.
5. The MicroPulse TLT treatment was performed.

Analgosedation was performed by the in-house anesthesia team under monitored anesthesia. First, an intravenous bolus of 50 mg fentanyl was administered followed by a bolus of thiopental sodium 0.5 g/20 ml two to three minutes later, which was adjusted to the patient's weight and renal function. Both forms of anesthesia were explained and offered to all patients.

MicroPulse TLT was delivered in a standardized fashion using 2′500 mW of power at a duty cycle of 31.3% with 60 seconds in the superior and 60 seconds in the inferior hemisphere, with each hemisphere receiving three 20-second sweeps. Two-percent Lidocaine gel (study group) or 2% methylcellulose (Methocel® 2%; OmniVision, Neuhausen, Switzerland) (control group) was used as a coupling agent. The footplate of the MicroPulse P3 probe was placed at the limbus with its “bunny ears” oriented toward the central cornea, which positioned the fiberoptic over the pars plana. Slight pressure of the revised MicroPulse P3 probe during the sweeping motion along the limbus ensured contact with the globe. Care was taken to avoid the three- and the nine-o'clock meridians as to not damage the long ciliary nerves, areas of scleral thinning, and sites of filtering blebs or glaucoma drainage devices. After the laser was applied, a fixed-combination ointment of tobramycin 3 mg/ml plus dexamethasone 1 mg/ml (Tobradex ointment; Alcon, Fort Worth, TX, USA) was applied to the eye. The eye was left unpatched.

The day after the procedure, patients were started on topical fixed-combination tobramycin 3 mg/ml plus dexamethasone 1 mg/ml (Tobradex eye drops; Alcon, Fort Worth, TX, USA) eye drops 5x/d for two weeks. The medical treatment was adapted from the standard of care treatment after cataract surgery. If cells or flare were still detected after two weeks, the drops were tapered over two additional weeks. Patients were directed to continue with their preoperative antiglaucoma medication regimen unless instructed otherwise. Medical hypotensive treatment was adjusted for each patient on every visit at the surgeon's discretion.

### 2.3. Baseline and Follow-Up Data Collection

Data were collected from the patients' medical records, including demographics, that is, age at surgery, diagnosis, gender, spherical equivalent, and mean defect on perimetry. Intraocular pressure (IOP), number of glaucoma medications, and best-corrected visual acuity (BCVA) were collected preoperatively and at 1 day, 1 week, and 1 month postoperatively. In addition, medical records were checked for adverse events at the postoperative time points. The pain level was recorded from medical records preoperatively, immediately after the treatment, and at 1 hour, 6 hours, 1 day, 1 week, and 1 month postoperatively. Pain was routinely recorded using a numeric rating pain scale from zero (no pain) to ten (unbearable pain) [20]. The numeric rating pain scale correlates positively with other measures of pain and, in the literature, showed sensitivity to treatments that are expected to affect pain [20]. For the 6-hour time point, the patients were contacted by phone, and all other pain levels where recoded while the patient was present in the operating theater, recovery unit, or outpatient department. Furthermore, the National Eye Institute Visual Function questionnaire was used to account for visual quality of life at baseline and one month postoperatively [21]. The questionnaire was distributed to glaucoma patients around the time the study was conducted before and after surgeries. Furthermore, data regarding prior incisional glaucoma surgery were collected from the medical records.

### 2.4. Statistical Analyses

Microsoft Excel for Macintosh Version 16.56 was used for data management, and IBM SPSS Statistics for Windows (International Business Machines Corporation (IBM), Armonk, NY, USA) version 26 was used for statistical analysis. Descriptive statistics were reported as mean ± SD for continuous variables and as absolute values and percentage for categorical variables. Preoperative and postoperative data and differences between the study and control group were compared using Student's *t*-test for equality of means (continuous variables) and chi-square test (categorical variables, Fisher's Exact Test). A *P* value of <0.05 was considered to be statistically significant.

## 3. Results

Eight eyes, four in the control group and four in the study group, were included in the study. The mean age at the time of the MicroPulse TLT was 78.3 ± 6.4 years. There was no difference between both study groups in regard to diagnosis, gender, eye, age at treatment, BCVA, IOP, number of meds, spherical equivalent, mean defect of static perimetry, pain level, or vision-related quality of life (see Table 1). No adverse events or serious adverse events occurred during the study period.

Data for pain level and the questionnaire were available for all patients at all time points. The reported pain level at baseline was statistically higher in the study group than the control group (0.5 vs. 0.0, *P*). Both groups reported mild or no pain, respectively. The reported pain level increased slightly but statistically significant in the study group immediately after the treatment (from 0.5 to 2.8, *p*=0.003; a score between 0 and 5 is considered mild pain). However, no difference was found at any later time point compared to baseline (see Table 2). In the control group, there was no change in pain level at any time point compared to baseline (see Table 2). No patient in either group required additional medication post-treatment due to pain.

The vision-related quality of life was similar in both groups and was not negatively impacted by MicroPulse TLT at 1 month after the treatment (see Table 3). At baseline, the NEI-VFQ score was 118.3 ± 11.0 in the study and 121.0 ± 14.1 in the control group (*p*=0.768) and at one month 120.5 ± 8.5 and 124.5 ± 18.2, respectively. The change between baseline and one month was not statistically significant (*p*=0.215 and *p*=0.188, respectively).

There was no statistically significant difference in regard to BCVA, IOP, or glaucoma medications between groups or within each group, not at baseline (see Table 1) and not at any subsequent time point compared to baseline (see Table 4). One patient had a prior incisional glaucoma surgery (i.e., trabeculectomy with Mitomycin C). The area of the filtering bleb was spared during MicroPulse TLT.

## 4. Discussion/Conclusion

The MicroPulse TLT procedures in the “Topical Plus” group in this study were performed as monitored anesthesia care. Historically, our Cyclo G6 Laser is located in the operating theater with standby anesthesia available for all glaucoma cases. This concept enables us even with MAC to provide all patients with immediate intravenous fentanyl for pain control, in case any form of topical anesthesia is not sufficiently controlling for pain. Although, there was a mild significant increase in the pain level of the study group compared to baseline, and compared to the analgosedation group, no patient in the study group requested fentanyl. The increase in pain level in the study group was only mild. A pain score equal to or smaller than 5 is considered to be mild (a pain score between 6 and 7 is considered moderate, and scores equal to or greater than 8 are considered severe pain). Furthermore, the increase in pain level in the study group was of short duration only; no increased pain level compared to baseline was reported at 1 hour or at any later time point in the “Topical Plus” group. The MicroPulse TLT treatment could be conducted sufficiently in all cases independently of the anesthesia protocol used. The pain level at baseline was statistically higher in the study group than the control group (0.5 vs. 0.0, *p*=0.024). Thus, the results support sufficient pain control under the proposed “Topical Plus” anesthesia protocol. The pain levels at later time points (i.e., 1 week and 1 month) are very consistent with preoperative values. This is confirming the reliability of the used pain scale.

All treated eyes had good visual acuity (VA; i.e., ≥20/60) and early to moderate glaucomatous visual defects in static perimetry. Although sample size was small and follow-up was only 1 month, MicroPulse TLT did not negatively impact VA and affirms the results from previous studies, which found no negative impact by MicroPulse TLT on VA in eyes with good central vision (see Table 4) [22, 23].

No change in IOP or reduction in glaucoma medications was achieved, nor expected, due to the short follow-up period and because eyes were postoperatively treated with topical steroids for 2 to 4 weeks (see Table 4). IOP decrease after MicroPulse TLT is expected after discontinuation of topical steroids and after a potential steroid response would fade out, which is >4 or >6 weeks (depending on the length of topical steroid treatment) after the treatment. The study was not designed to evaluate IOP reduction after MicroPulse TLT.

In the literature, most often the use of subtenon's anesthesia, peribulbar, or retrobulbar blocks has been reported [12, 23–48]. Only a few studies reported the use of general anesthesia [11, 25, 44, 49–51]. All studies which performed MicroPulse TLT in children used general anesthesia [52, 53]. To the best of our knowledge, there has not been a randomized controlled trial performed comparing different forms of anesthesia for sufficient pain control for MicroPulse TLT. No study systematically used an analog pain scale and vision-related quality of life questionnaires and compared results at baseline with postoperative values at various time points. Only one study reported pain in the first hours after the procedure [43]. In some countries, the use of propofol for sedation is more common than thiopental. Both drugs are of short action, provide a similar recovery time, and have a comparable safety profile, which make them favorable for short procedures performed under sedation [54]. Patients recalled discomfort on the injection side more often after propofol than after thiopental [54]. Apnea was reported to be more common after propofol compared to thiopental [55]. This can be a disadvantage as ventilation cannot be performed at the same time as the procedure at an eye.

Prolonged or persistent mydriasis after MicroPulse TLT has been described [39]. It is usually mild and often reversible with time. The proposed mechanism is damage by laser energy to the long ciliary nerves. Hence, surgeons usually spare the 3 and 9 o'clock positions including a safety zone. A block, general anesthesia, and analgosedation result in some degree of inevitable cyclorotation of the globe. Cyclorotation can inadvertently cause damage of the long ciliary nerves due to direct exposure by laser energy. Hence, it is preferable to mark the 3 and 9 o'clock positions prior to anesthesia and while the patient is in sitting position. The “Topical Plus” anesthesia should not, or may lessen, cause the occurrence of cyclorotation and, thus, should result in less damage to the long ciliary nerves and postop mydriasis.

The limitation of this study is the retrospective design and the small sample size. The study was intended as a first proof-of-concept. However, the very similar results of pain control in both study groups, that is, “Topical Plus” vs. analgosedation, confirm our personal impression that the “Topical Plus” protocol enables adequate anesthesia for MicroPulse TLT. Another limitation is that one study arm is awake during the MicroPulse TLT while the other arm is not. Hence, there is potential confounding bias of the reported pain level immediately after the laser procedure by the experienced pain level during the laser procedure in the Topical Plus study arm. This bias is impossible to avoid. It is also not possible to record pain levels during the procedure in the analgosedation arm. Different coupling agents have been used in the two study arms, that is, lidocaine gel vs. methylcellulose. As anesthesia with 2% lidocaine gel was to be investigated in the study group and compared to analgosedation, 2% methylcellulose was not additionally applied to the eyes in the study group given that the surface of these eyes was already well covered with 2% lidocaine gel throughout the entire laser procedure. There is evidence for the importance of a coupling agent; however, there is no proof that the physical and chemical compound properties of different coupling agents (lidocaine gel vs. methylcellulose bot also BSS) have a clinically significant divergent effect of laser conduction and efficacy [19]. Different pressures with which the P3 probe is applied to the eye could result in different pain levels. Although the Rev 2 P3 laser probe has no protruding laser fiber in comparison to the original version of the probe, which should minimize this effect. During MicroPulse TLT, the surgeon applies very carefully just as much pressure as needed to assure that the laser probe is in contact with the globe during the procedure but not more, for example, the laser probe is not pushing the globe back into the fat tissue within the orbit. All MicroPulse TLT procedures in this study were performed by the same surgeon who has a long experience with this technique. Thus, there should be only little variation of the pressure of the laser probe applied to the globes between the different study eyes.

As a next step, a study with a larger sample size, ideally prospective and randomized, comparing different forms of anesthesia (e.g., analgosedation, subconjunctival anesthesia, peribulbar, and retrobulbar blocks) with the proposed “Topical Plus” protocol is desired. The analog pain scale at different time points (e.g., at baseline and postoperative at 1 h, 6 h, 12 h, 24 h, 1 week, and 1 month) and the evaluation of patient comfort (e.g., QoL questionnaires and vision-related QoL questionnaires) should be incorporated in the randomized controlled trial. Because we found an increase in pain level immediately after the procedure in the study arm but not at 1 h or later, ideally pain levels should be monitored more closely in the immediate period after the procedure, for example additionally at 15, 30, and 45 minutes after the procedure. The study population consisted exclusively of Caucasians from Switzerland. Thus, it is unknown if the results are generalizable to a multiethnical population outside of Switzerland. A follow-up study should ideally include patients of various ethnicities and should include eyes with different levels of conjunctival pigmentation as the level of pigmentation could potentially influence pain sensation by transscleral laser treatment. Some authors reported higher odds of prolonged inflammation in heavily pigmented eyes by transscleral laser treatment [56]. This could be due to the high energy delivered in this study, as the laser was delivered in a “stop-and-go” pattern (i.e., it was held in place for 10 seconds before being moved to the adjacent section of perilimbal conjunctiva) for 120 to 360 seconds. Furthermore, MicroPulse TLT is performed with different treatment times. It is unknown if the findings from this study are transferable to longer treatment times, that is., >60 sec. per hemisphere.

The favorable pain control with the “Topical Plus” anesthesia protocol enables MicroPulse TLT to be delivered with sufficient pain control for the patient to remain comfortable during and post-treatment. The “Topical Plus” protocol does not require monitored anesthesia care; hence, no standby anesthesia team is necessary. Thus, MicroPulse TLT can be performed independently from an inpatient or outpatient surgical center. This was an initial proof-of-concept study only with a very limited number of patients. Thus, the results must be judged carefully, and it is unknown whether they can be generalized. However, a sufficient pain control with topical anesthesia only would enable glaucoma specialists to perform MicroPulse TLT safely in an office setting. The advantage for the patient would be a more comfortable treatment than a procedure in a surgical setting which often results in increased anxiety levels. The positive results of the proof-of-concept study encourage us to plan a prospective, randomized-controlled trial with a larger sample size. Ideally, the study should be conducted as a multicenter study to enroll patients of different ethnicities and ages.

## 5. Consent

All study subjects gave written informed consent.

## Figures and Tables

**Table 1:** Demographics.

	All (*n* = 8)	Topical plus (*n* = 4)	Analgosedation (*n* = 4)	*P* value
Diagnosis				0.486
POAG	4	3	1	
PEXG	4	1	3	

Gender				0.429
Male	2	2	0	
Female	6	2	4	

Eye				0.143
RE	3	0	3	
LE	5	4	1	

Age at treatment (years)	78.3 ± 6.4	73.0 ± 4.3	83.6 ± 1.3	0.221
BCVA at screening (logMAR)	0.3 ± 0.3	0.1 ± 0.1	0.4 ± 0.4	0.103
IOP at screening (mmHg)	17.1 ± 2.7	17.5 ± 3.3	16.8 ± 2.4	0.490
Meds at screening (*n*)	2.6 ± 1.3	2.8 ± 1.9	2.5 ± 0.6	0.140
Spherical equivalent (*D*)	0.25 ± 1.78	0.56 ± 1.83	−0.06 ± 1.94	0.903
Mean defect at screening (dB)	5.5 ± 3.6	3.8 ± 3.5	7.2 ± 3.2	0.716
Pain scale at screening (0,…, 10)	0.3 ± 0.8	0.5 ± 1.0	0.0 ± 0.0	0.024
NEI-VFQ at screening (45,…, 256)	119.6 ± 11.8	118.3 ± 11.0	121.0 ± 14.1	0.257

(POAG = primary open-angle glaucoma, PEXG = pseudoexfoliative glaucoma, RE = right eye, LE = left eye, BCVA = best corrected visual acuity, LogMAR = logarithm of the minimum angle Of resolution, mmHg = millimeter of mercury, D = diopter, dB = decibel, and NEI-VFQ = National Eye Institute Visual Function Questionnaire).

**Table 2:** Change in reported pain levels.

	Topical plus	*P* value	Analgosedation	*P* value	*P* value between groups
At screening	0.5 ± 1.0	—	0.0 ± 0.0	—	0.356
Immediately post-treatment	2.8 ± 1.5	0.003	0.0 ± 0.0	^a^	0.010
1 h post-treatment	0.8 ± 1.5	0.391	0.0 ± 0.0	^a^	0.356
6 h post-treatment	0.0 ± 0.0	0.391	0.0 ± 0.0	^a^	^a^
1 D post-treatment	0.0 ± 0.0	0.391	0.0 ± 0.0	^a^	^a^
1 W post-treatment	0.0 ± 0.0	0.391	0.0 ± 0.0	^a^	^a^
1 M post-treatment	0.0 ± 0.0	0.391	0.0 ± 0.0	^a^	^a^

h = hour(s), D = day, W = week, and M = month. ^a^t cannot be computed because the standard deviations of both groups are 0.

**Table 3:** Change in vision-related quality of life.

	Topical plus	*P* value	Analgosedation	*P* value	*P* value between groups
NEI-VFQ at screening	118.3 ± 11.0	—	121.0 ± 14.1	—	0.768
NEI-VFQ 1 M post-treatment	120.5 ± 8.5	0.215	124.5 ± 18.2	0.188	0.704

NEI-VFQ = National Eye Institute Visual Function Questionnaire; M = month.

**Table 4:** Change in visual acuity, intraocular pressure, and medication.

	Topical plus	*P* value	Analgosedation	*P* value	*P* value between groups
BCVA at screening [logMAR]	0.1 ± 0.1	—	0.4 ± 0.4	—	0.246
BCVA 1 D post-treatment [logMAR]	0.0 ± 0.1	0.500	0.5 ± 0.1	0.671	0.248
BCVA 1 W post-treatment [logMAR]	0.1 ± 0.2	0.500	0.4 ± 0.4	0.934	0.340
BCVA 1 M post-treatment [logMAR]	0.0 ± 0.1	0.206	0.3 ± 0.3	0.418	0.105
IOP at screening (mmHg)	17.5 ± 3.3	—	16.8 ± 2.4	—	0.725
IOP 1 D post-treatment (mmHg)	16.0 ± 8.5	0.930	25.7 ± 6.6	0.225	0.244
IOP 1 W post-treatment (mmHg)	19.5 ± 0.7	0.874	18.8 ± 5.9	0.480	0.873
IOP 1 M post-treatment (mmHg)	17.0 ± 7.2	0.861	17.5 ± 2.4	0.225	0.899
Meds at screening	2.8 ± 1.9	—	2.5 ± 0.6	—	0.809
Meds 1 D post-treatment	2.8 ± 1.9	^a^	2.0 ± 1.2	0.182	0.524
Meds 1 W post-treatment	2.8 ± 1.9	^a^	2.0 ± 1.2	0.182	0.524
Meds 1 M post-treatment	2.8 ± 1.9	^a^	2.0 ± 1.1	0.182	0.524

^a^ *t* cannot be computed because the standard error of the differences is 0. h = hour(s), D = day, W = week, M = month, BCVA = best corrected visual acuity, logMAR = Logarithm of the Minimum Angle of Resolution, IOP = intraocular pressure, and mmHg = millimeter of mercury.

## Data Availability

Data are available on request.
